# Planned mode of delivery and neonatal outcomes in pregnancies complicated by late-onset fetal growth restriction: a retrospective cohort study

**DOI:** 10.1007/s00404-026-08357-8

**Published:** 2026-03-02

**Authors:** Misgav Rottenstreich, Eran Ashwal, Amal Yousef, Bryon DeFrance, Jon F. R. Barrett, Hen Y. Sela

**Affiliations:** 1 Division of Maternal-Fetal Medicine, Department of Obstetrics and Gynecology, McMaster University, Hamilton, ON Canada; 2 Department of Obstetrics & Gynecology, Shaare Zedek Medical Center, Affiliated with the Hebrew University School of Medicine, Jerusalem, Israel; 3 Division of Maternal-Fetal Medicine, Department of Obstetrics and Gynecology, Hamilton Health Sciences, McMaster University, 1200 Main St. West, Hamilton, ON L8N 3Z5 Canada

**Keywords:** FGR, Fetal growth restriction, Induction of labor, Cesarean delivery, Neonatal outcomes, Pregnancy complications, Obstetric management, Neonatal morbidity, Perinatal outcomes

## Abstract

**Background:**

Fetal growth restriction (FGR) is a major contributor to perinatal morbidity and mortality. While guidelines address timing of delivery, the optimal mode—induction of labor (IOL) versus planned cesarean delivery (CD)—remains uncertain.

**Objective:**

To evaluate the association between planned mode of delivery and neonatal outcomes in pregnancies complicated by late onset FGR (LOFGR).

**Study Design:**

We conducted a retrospective cohort study at a tertiary Canadian center (2017–2022). Singleton pregnancies with LOFGR (> 34 weeks’ gestation), defined by Society for Maternal–Fetal Medicine (SMFM) criteria, were eligible if the last ultrasound was within 14 days of delivery. Exclusions included spontaneous labor, delivery < 34 weeks, and contraindications to labor. Planned mode of delivery (IOL vs CD) was the exposure. Outcomes were classified as severe (perinatal death, 5-min Apgar < 4, umbilical arterial pH < 7.05, base deficit ≥ 12 mmol/L, hypoxic-ischemic encephalopathy/therapeutic hypothermia, grade III–IV intraventricular hemorrhage, necrotizing enterocolitis, sepsis, or invasive ventilation > 24 h) or moderate (NICU stay > 72 h, Apgar 4–6, pH 7.05–7.10, non-invasive respiratory support > 6–12 h, transient tachypnea, or brief resuscitation). Multivariable logistic regression adjusted for confounders. A prespecified subgroup applied the ISUOG criteria.

**Results:**

Of 12,270 deliveries, 1,143 (9.3%) met SMFM criteria for LOFGR; 869 were eligible (192 planned CD, 677 IOL). Severe outcomes and moderate outcomes were more frequent after CD (23.4% vs 16.7%; *p* = 0.03 and 42.2% vs 31.2%; *p* < 0.01, respectively). IOL was associated with lower adjusted risk of severe outcomes (aOR 0.35; 95% CI 0.19–0.67) and moderate outcomes (aOR 0.43; 95% CI 0.24–0.76). Results were consistent using ISUOG criteria (aOR 0.33; 95% CI 0.17–0.62 and aOR 0.44; 95% CI 0.25–0.79, respectively) About 20% of induced patients required intrapartum CD.

**Conclusions:**

IOL was associated with reduced severe and moderate neonatal morbidity compared with planned CD. IOL represents a safe alternative when intrapartum surveillance and timely operative delivery are available.

**Supplementary Information:**

The online version contains supplementary material available at 10.1007/s00404-026-08357-8.

## What does this study adds to the clinical work

**Table:** 

In pregnancies complicated by late-onset fetal growth restriction beyond 34 weeks, induction of labor was associated with lower rates of severe and moderate neonatal morbidity compared with planned cesarean delivery. When close intrapartum surveillance and timely operative intervention are available, induction represents a safe and reasonable alternative to planned cesarean delivery.	

## Introduction

Fetal growth restriction (FGR) is one of the most common obstetric complications and a major contributor to perinatal morbidity and mortality [1–3]. It is defined as a pathological condition in which the fetus fails to achieve its genetically determined growth potential.[4, 5]

Fetuses with FGR are at substantially increased risk of stillbirth [6, 7] and short-term morbidities, including meconium aspiration, hypoglycemia, polycythemia, and low Apgar scores [3, 8, 9]. In the intermediate term, FGR is associated with neurodevelopmental sequelae such as cerebral palsy, learning disorders, and cognitive impairment [10, 11]. Long-term consequences extend into adulthood, with higher rates of cardiovascular disease, hypertension, diabetes, hyperlipidemia, and obesity [12, 13].

Perinatal outcomes among FGR fetuses largely depend on the severity of growth restriction [14]. Early recognition, careful surveillance, and timely delivery are central to improving outcomes [15, 16]. Current guidelines, including those of the Society for Maternal–Fetal Medicine (SMFM) and the International Society of Ultrasound in Obstetrics and Gynecology (ISUOG), provide recommendations regarding diagnosis, surveillance, and timing of delivery in FGR [17, 18]. However, both organizations acknowledge the lack of robust evidence to guide decisions on the optimal mode of delivery for these pregnancies.

Whether induction of labor (IOL) or planned cesarean delivery (CD) confers greater neonatal benefit remains uncertain, with existing data being limited and inconsistent [19–22]. Addressing this knowledge gap is critical, given the high clinical stakes and the potential to directly influence delivery management in FGR.

Therefore, the objective of this study was to evaluate the impact of the planned mode of delivery on adverse neonatal outcomes in pregnancies complicated by late onset FGR (LOFGR, > 34 weeks’ gestation).

## Materials and methods

### Study design and population

We conducted a retrospective cohort study at a university-affiliated tertiary care center in Ontario, Canada, including singleton pregnancies complicated by fetal growth restriction (FGR) between January 2017 and December 2022. FGR was defined according to the Society for Maternal–Fetal Medicine (SMFM) criteria as an estimated fetal weight (EFW) and/or abdominal circumference (AC) below the 10th percentile for gestational age [20], based on Hadlock reference charts [23, 24].To ensure contemporaneous assessment of fetal condition, only pregnancies with a documented ultrasound examination within 14 days prior to delivery were eligible.

Exclusion criteria were multifetal gestation, delivery before 34 + 0 weeks’ gestation (as management and delivery practices prior to this gestational age have been established [18], spontaneous onset of labor, known fetal structural or genetic anomalies, intrauterine fetal demise prior to labor, pregnancy termination, contraindications to labor (non-vertex presentation, more than two prior cesarean deliveries, placenta previa or vasa previa, prior classical cesarean delivery or other contraindicating uterine surgery, known uterine rupture risk, active genital herpes infection, any condition requiring immediate cesarean delivery at admission), or delivery outside the hospital setting.

### Data sources and variables

Data were extracted from a prospectively maintained electronic medical record system and included maternal demographic characteristics, obstetric history, ultrasound and Doppler findings, labor and delivery details, and neonatal outcomes. All data were de-identified prior to analysis. The study was approved by the McMaster University Research Ethics Board (IRB #15003), with waiver of informed consent due to the retrospective design.

### Exposure

The exposure of interest was the planned mode of delivery, classified as induction of labor (IOL) or planned cesarean delivery (CD). Planned CD was defined as a cesarean delivery scheduled prior to the onset of labor or initiation of induction. Patients who entered spontaneous labor were excluded.

### Outcomes

The primary outcome was a composite severe adverse neonatal outcome, defined as the occurrence of one or more of the following: perinatal death (neonatal death within 28 days), umbilical arterial pH < 7.05 or base deficit ≥ 12 mmol/L, 5-min Apgar score < 4, moderate-to-severe hypoxic-ischemic encephalopathy or receipt of therapeutic hypothermia, grade III–IV intraventricular hemorrhage, Bell stage ≥ II necrotizing enterocolitis, culture-proven neonatal sepsis, need for invasive mechanical ventilation > 24 h, or persistent pulmonary hypertension of the newborn requiring advanced therapy.

The secondary outcome was a composite moderate adverse neonatal outcome, defined as one or more of the following: NICU admission > 72 h, 5-min Apgar score 4–6, umbilical arterial pH 7.05–7.10, need for non-invasive respiratory support (continuous positive airway pressure or high-flow nasal cannula > 6–12 h), transient tachypnea of the newborn, or neonatal resuscitation limited to bag-mask ventilation or brief positive-pressure ventilation.

Individual components of each composite were reported separately. Given heterogeneity in clinical significance and susceptibility to practice variation across components, adjusted analyses were prespecified only for individual outcomes with sufficient event counts.

### Subgroup analysis

A planned subgroup analysis was conducted using the International Society of Ultrasound in Obstetrics and Gynecology (ISUOG) definition of FGR [18], endorsed by the Society of Obstetricians and Gynaecologists of Canada [25]. This definition includes either AC or EFW < 3rd percentile, or at least two of the following: AC or EFW < 10th percentile, downward crossing of more than two quartiles on growth curves, cerebroplacental ratio (CPR) < 5th percentile, or umbilical artery pulsatility index (UA-PI) > 95th percentile.

### Statistical analysis

Baseline characteristics were summarized using means with standard deviations or medians with interquartile ranges for continuous variables, and proportions for categorical variables. Group comparisons were performed using Student’s *t*-test or Mann–Whitney *U* test for continuous variables and Chi-square or Fisher’s exact test for categorical variables, as appropriate.

Covariates were selected a priori based on clinical relevance and causal considerations, rather than statistical significance alone. These included maternal age, parity, prior cesarean delivery, obesity (BMI > 30), fertility treatment, diabetes, hypertensive disorders, abnormal Doppler findings, and gestational age at last ultrasound.

Multivariable logistic regression models were used to estimate adjusted odds ratios (aORs) with 95% confidence intervals (CIs) for the association between planned mode of delivery and neonatal outcomes. Gestational age at birth was not included in the primary adjusted models due to its potential role as a mediator on the causal pathway between delivery planning and neonatal outcomes. Sensitivity analyses were performed with gestational age at birth included to assess the robustness of findings.

To further address confounding by indication, a propensity score–adjusted sensitivity analysis was performed. The propensity score for induction of labor versus planned cesarean delivery was estimated using logistic regression incorporating maternal, obstetric, and Doppler characteristics determined a priori. The propensity score was then included as an adjustment variable in logistic regression models evaluating neonatal outcomes.

Given heterogeneity within the composite, adjusted analyses were also performed for key individual components with sufficient event counts.

Outcomes with very low or zero event counts were compared using Fisher’s exact test and were not modeled using multivariable regression due to risk of separation and unstable estimates.

### Missing data

The completeness of data was high for most key variables (Umbilical artery Doppler indices were available for 98% of pregnancies, middle cerebral artery Doppler indices for 63%, and cerebroplacental ratio for 61%. Umbilical cord blood gas measurements were available for 95% of neonates, and neonatal intensive care unit outcomes for 96%). Given the overall low proportion of missing data for primary outcomes and most covariates, analyses were conducted using a complete-case approach. Doppler variables with higher rates of missingness primarily reflected variation in clinical practice and ultrasound protocols over the study period. To minimize potential bias, Doppler parameters were incorporated selectively into multivariable and propensity score models, and sensitivity analyses were performed to assess the robustness of the findings.

All statistical tests were two-sided, with *p* < 0.05 considered statistically significant. Analyses were performed using SPSS Statistics version 25 (IBM Corp., Armonk, NY). Results are reported in accordance with STROBE guidelines for observational studies [26].

## Results

### Study population

**Fig. 1:**
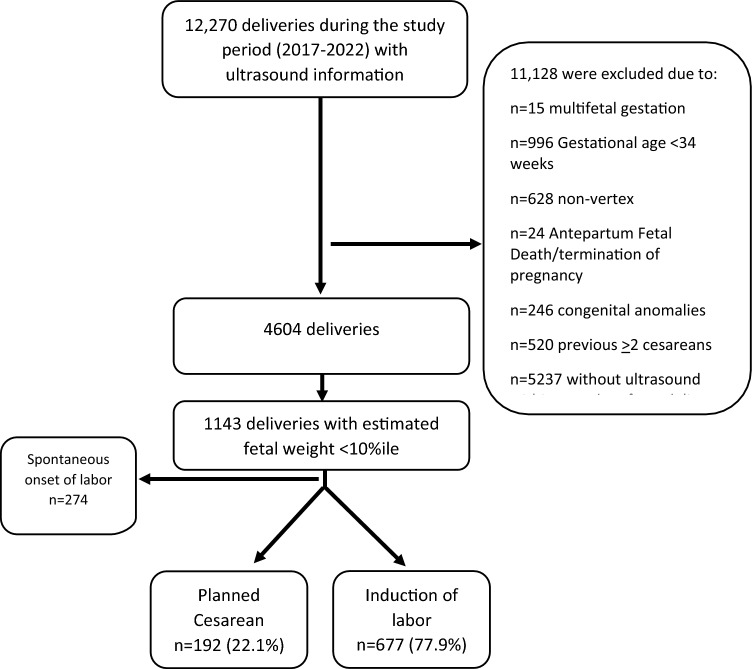
Study flow chart

During the study period, 12,270 deliveries with available ultrasound data were identified. After applying inclusion and exclusion criteria, 1,143 pregnancies were classified as LOFGR based on the SMFM definition. Of these, 869 cases involved either IOL or planned CD and were included in the final analysis. Among them, 192 pregnancies (22.1%) were managed with planned CD and 677 (77.9%) with IOL (Fig. 1).

### Demographic and obstetric characteristics

Maternal and obstetric characteristics are summarized in Table 1. Compared with the planned CD group, patients undergoing IOL were younger, more frequently nulliparous, and less likely to have obesity (BMI > 30) or a history of fertility treatment. Abnormal cerebroplacental ratio (CPR) was less prevalent in the IOL group, and deliveries occurred at more advanced gestational ages.

**Table 1:** Maternal and pregnancy characteristics (SMFM definition)

	Planned cesarean *n* = 192	Induction of labor *n* = 677	*p*-value
Maternal age, years	32.5 ± 4.9	30.6 ± 5.5	< 0.01
Maternal age ≥ 35 years	70 (36.5%)	162 (23.9%)	< 0.01
Gravida	2.6 ± 1.7	2.4 ± 1.7	0.27
Parity	0.9 ± 0.9	0.8 ± 1.2	0.41
Nulliparity	59 (30.7%)	377 (55.7%)	< 0.01
Obesity (BMI ≥ 30)	61 (32.4%)	155 (23.4%)	0.01
Fertility Treatments	24 (12.5%)	52 (7.7%)	0.04
Smoking	24 (12.5%)	93 (13.7%)	0.66
Diabetes (pregestational and gestational)	39 (20.3%)	100 (14.8%)	0.06
Pre-existing diabetes	11 (5.7%)	22 (3.2%)	0.11
Preeclampsia	11 (5.7%)	33 (4.9%)	0.63
Gestational age at last ultrasound	34.4 ± 3.1	35.6 ± 3.4	< 0.01
BPP in last ultrasound < 8/8	52 (27.7%)	164 (24.7%)	0.41
Abnormal umbilical artery PI^a^	36 (19.4%)	94 (14.2%)	0.08
Abnormal MCA_PI^b^	20 (18%)	67 (14.8%)	0.40
Abnormal CPR^c^	69 (62.7%)	225 (49.9%)	0.02
Oligohydramnios ^d^	14 (7.3%)	76 (11.4%)	0.11
Gestational age at birth, weeks	37.1 ± 1.4	37.9 ± 1.3	< 0.01
Gestational age at birth < 37 weeks	63 (32.8%)	86 (12.7%)	< 0.01

Data are mean ± standard deviation; number (%)

*BMI* body mass index, *AC* abdominal circumference, *BPP* biophysical profile, *CPR* Cerebral Placental Ratio (MCA-PI/UA-PI), *MCA* middle cerebral artery, *PI* pulsatility index, *UA *umbilical artery

^a^UA PI > 95 percentile according to Acharya G, Wilsgaard T, Berntsen GKR, Maltau JM, Kiserud T. Reference ranges for serial measurements of umbilical artery Doppler indices in the second half of pregnancy. Am J Obstet Gynecol. 2005 Mar;192(3):937–44

^b^MCA PI < 5 percentile according to Bahlmann F, Reinhard I, Krummenauer F, Neubert S, Macchiella D, Wellek S. Blood flow velocity waveforms of the fetal middle cerebral artery in a normal population: reference values from 18 to 42 weeks of gestation. J Perinat Med 2002;30:490–501. https://doi.org/10.1515/JPM.2002.077

^c^CPR < 5 percentile

^d^Oligohydramnios—maximal vertical pocket < 2 cm and/or Amniotic Fluid Index < 5 cm

### Perinatal outcomes

Perinatal outcomes are presented in Table 2.

- Severe composite outcome: Severe adverse neonatal outcomes occurred more frequently in the planned CD group compared with the IOL group (23.4% vs. 16.7%; *p* = 0.03).
- Moderate composite outcome: Moderate adverse neonatal outcomes were also more common in the planned CD group (42.2% vs. 31.2%; *p* < 0.01).
- Individual components: Compared with planned CD, IOL was associated with lower rates of mechanical ventilation (13.6% vs. 22.9%; *p* < 0.01), prolonged NICU admission > 72 h (18.9% vs. 27.1%; *p* = 0.01), respiratory distress syndrome (0.3% vs. 2.1%; *p* = 0.01), transient tachypnea (5.8% vs. 12.5%; *p* < 0.01), 5-min Apgar 5–7 (2.8% vs. 6.8%; *p* = 0.01), and need for neonatal resuscitation (13.2% vs. 22.4%; *p* < 0.01). However, umbilical arterial pH < 7.05 (2.9% vs 0.0%, *p* = 0.04) and base deficit ≥ 12 mmol/L (4.7% vs 0.6%, *p* = 0.01) were more frequent in the IOL group than in the planned cesarean delivery group.
- Among patients undergoing IOL, 20.7% required an unplanned CD, with 15.1% performed for non-reassuring fetal heart rate tracings.

**Table 2:** Perinatal outcomes of late onset FGR pregnancies as per the SMFM definition who underwent induction of labor as compared to planned cesarean delivery

	Planned cesarean *n* = 192	Induction of labor *n* = 677	*p*-value
Composite severe adverse neonatal outcome^a^	45 (23.4%)	113 (16.7%)	0.03
Perinatal death	0 (0%)	2 (0.3%)	0.45
5-min Apgar < 4	0 (0%)	4 (0.6%)	0.29
Arterial cord blood pH < 7.05	0 (0%)	16 (2.9%)	0.04
Base deficit ≥ 12 mmol/L	1 (0.6%)	30 (4.7%)	0.01
Intracranial hemorrhage	0 (0%)	0 (0%)	N/A
Sepsis	0 (0%)	1 (0.1%)	0.59
Necrotizing enterocolitis	0 (0%)	0 (0%)	N/A
Ventilation	44 (22.9%)	92 (13.6%)	< 0.01
Composite moderate adverse neonatal outcome^b^	81 (42.2%)	211 (31.2%)	< 0.01
Prolong length of stay NICU (> 72 h)	52 (27.1%)	128 (18.9%)	0.01
Respiratory distress syndrome	4 (2.1%)	2 (0.3%)	0.01
Transient tachypnea	24 (12.5%)	39 (5.8%)	< 0.01
Arterial cord blood pH 7.05 to 7.10	4 (2.3%)	28 (4.4%)	0.21
5-min Apgar—5–7	13 (6.8%)	19 (2.8%)	0.01
Neonatal Resuscitation (first 30 min of life only)	43 (22.4%)	89 (13.2%)	< 0.01
Other outcomes			
CD unplanned	–	140 (20.7%)	N/A
CD unplanned NRFHR	–	102 (15.1%)	N/A
Operative vaginal delivery	–	2 (0.3%)	N/A
Postpartum hemorrhage	10 (5.2%)	41 (6.1%)	0.66

Data are mean ± standard deviation; number (%)

*CD* Cesarean Delivery, *NICU* neonatal intensive care unit, *NRFHR* non-reassuring fetal heart rate

^a^Defined as the presence of at least one of the following: perinatal death (neonatal death within 28 days), umbilical arterial pH < 7.05 or base deficit ≥ 12 mmol/L, 5-min Apgar score < 4, moderate-to-severe hypoxic-ischemic encephalopathy or receipt of therapeutic hypothermia, grade III–IV intraventricular hemorrhage, Bell stage ≥ II necrotizing enterocolitis, culture-proven neonatal sepsis or need for invasive mechanical ventilation > 24 h

^b^Defined as at least one of the following: neonatal intensive care unit (NICU) admission > 72 h, 5-min Apgar score 4–6, umbilical arterial pH 7.05–7.10, need for non-invasive respiratory support (continuous positive airway pressure or high-flow nasal cannula > 6–12 h) without intubation, transient tachypnea of the newborn, or neonatal resuscitation limited to bag-mask ventilation or brief positive-pressure ventilation in the delivery room

### Multivariable logistic regression

Results of multivariable logistic regression are summarized in Table 3. After adjustment for maternal and obstetric covariates, IOL was associated with a significantly lower risk of the severe composite outcome compared with planned CD (aOR 0.35; 95% CI 0.19–0.67; *p* = 0.01). Additionally, IOL was also independently associated with the moderate composite outcome (aOR 0.43; 95% CI 0.24–0.76; *p* = 0.04).

**Table 3:** Adjusted OR and 95% confidence intervals (CI) of composite severe^a^ and moderate^b^ adverse neonatal outcome^a^ among late onset FGR fetuses as per the SMFM definition who underwent induction of labor as compared to planned cesarean delivery (reference group—planned cesarean delivery)

	*p*-value	aOR (95% CI)
Composite severe adverse neonatal outcome^a^	0.01	0.35 (0.19–0.67)
Composite moderate adverse neonatal outcome^b^	0.04	0.43 (0.24–0.76)

Adjusted for maternal age, nulliparity, previous cesarean delivery, obesity, fertility treatments, abnormal CPR (Cerebral Placental Ratio)

*FGR* Fetal Growth Restriction

^a^Defined as the presence of at least one of the following: perinatal death (neonatal death within 28 days), umbilical arterial pH < 7.05 or base deficit ≥ 12 mmol/L, 5-min Apgar score < 4, moderate-to-severe hypoxic-ischemic encephalopathy or receipt of therapeutic hypothermia, grade III–IV intraventricular hemorrhage, Bell stage ≥ II necrotizing enterocolitis, culture-proven neonatal sepsis or need for invasive mechanical ventilation > 24 h

^b^Defined as at least one of the following: neonatal intensive care unit (NICU) admission > 72 h, 5-min Apgar score 4–6, umbilical arterial pH 7.05–7.10, need for non-invasive respiratory support (continuous positive airway pressure or high-flow nasal cannula > 6–12 h) without intubation, transient tachypnea of the newborn, or neonatal resuscitation limited to bag-mask ventilation or brief positive-pressure ventilation in the delivery room

In adjusted analyses, IOL was associated with lower odds of invasive ventilation (aOR 0.29; 95% CI 0.15–0.56; *p* < 0.01) and prolonged NICU admission (aOR 0.45; 95% CI 0.24–0.83; *p* = 0.01), while differences in rare outcomes were not modeled due to low event counts.

**Table 4:** Perinatal outcomes of late onset FGR pregnancies as per the ISUOG definition who underwent induction of labor as compared to planned cesarean delivery

	Planned cesarean *n* = 155	Induction of labor *n* = 568	*p*-value
Composite severe adverse neonatal outcome^a^	42 (27.1%)	97 (17.1%)	< 0.01
Perinatal death	0 (0%)	2 (0.4%)	0.46
5-min apgar < 4	0 (0%)	4 (0.7%)	0.30
Arterial cord blood pH < 7.05	0 (0%)	11 (2%)	0.09
Base deficit ≥ 12 mmol/L	1 (0.7%)	23 (4.3%)	0.04
Intracranial hemorrhage	0 (0%)	0 (0%)	N/A
Sepsis	0 (0%)	0 (0%)	N/A
Necrotizing enterocolitis	0 (0%)	0 (0%)	N/A
Ventilation	41 (26.5%)	82 (14.4%)	< 0.01
Composite moderate adverse neonatal outcome^b^	70 (45.2%)	188 (33.1%)	0.01
Prolong length of stay NICU (> 72 h)	44 (28.4%)	114 (20.1%)	0.03
Respiratory distress syndrome	4 (2.6%)	2 (0.4%)	0.01
Transient tachypnea	21 (13.5%)	34 (6%)	< 0.01
Arterial cord blood pH 7.05–7.10	4 (2.9%)	25 (4.6%)	0.35
5-min Apgar—5 to 7	12 (7.7%)	17 (3%)	0.01
Neonatal resuscitation (first 30 min of life only)	40 (25.8%)	79 (14%)	< 0.01
Other outcomes			
CD unplanned	–	122 (21.5%)	N/A
CD unplanned NRFHR	–	87 (15.3%)	N/A
Operative vaginal delivery	–	0 (0%)	N/A
Postpartum hemorrhage	9 (5.8%)	30 (5.3%)	0.80

Data are mean ± standard deviation; number (%)

*CD* Cesarean Delivery, *NICU* neonatal intensive care unit, *NRFHR* non-reassuring fetal heart rate

^a^Defined as the presence of at least one of the following: perinatal death (neonatal death within 28 days), umbilical arterial pH < 7.05 or base deficit ≥ 12 mmol/L, 5-min Apgar score < 4, moderate-to-severe hypoxic-ischemic encephalopathy or receipt of therapeutic hypothermia, grade III–IV intraventricular hemorrhage, Bell stage ≥ II necrotizing enterocolitis, culture-proven neonatal sepsis or need for invasive mechanical ventilation > 24 h

^b^Defined as at least one of the following: neonatal intensive care unit (NICU) admission > 72 h, 5-min Apgar score 4–6, umbilical arterial pH 7.05–7.10, need for non-invasive respiratory support (continuous positive airway pressure or high-flow nasal cannula > 6–12 h) without intubation, transient tachypnea of the newborn, or neonatal resuscitation limited to bag-mask ventilation or brief positive-pressure ventilation in the delivery room

In propensity score–adjusted sensitivity analyses accounting for maternal, obstetric, and Doppler characteristics, IOL remained independently associated with a lower risk of the severe composite adverse neonatal outcome (aOR 0.34; 95% CI 0.18–0.66; *p* = 0.01) and the moderate composite outcome (aOR 0.51; 95% CI 0.29–0.90; *p* = 0.02) compared with planned cesarean delivery. Effect estimates were consistent with the primary multivariable models, supporting the robustness of the findings (Supplementary Table 1).

### Planned subgroup analysis (ISUOG definition of FGR)

A subgroup analysis was conducted among pregnancies meeting the ISUOG definition of FGR (Table 4). In total, 723 pregnancies were eligible, including 155 (21.4%) managed with planned CD and 568 (78.6%) with IOL.

- Severe composite outcome: Severe adverse neonatal outcomes were more frequent in the planned CD group compared with the IOL group (27.1% vs. 17.1%; *p* < 0.01).
- Moderate composite outcome: Moderate adverse neonatal outcomes were also more common after planned CD (45.2% vs. 33.1%; *p* = 0.01).
- Individual components: Similar to the SMFM-defined population, IOL was associated with lower rates of prolonged NICU stay, respiratory distress syndrome, transient tachypnea, Apgar 5–7, and neonatal resuscitation.

In multivariable logistic regression (Table 5), IOL was again associated with a significantly lower risk of the severe composite outcome (aOR 0.33; 95% CI 0.17–0.62; *p* = 0.01), and the moderate composite outcome (aOR 0.44; 95% CI 0.25–0.79; *p* < 0.01).

**Table 5:** Adjusted OR and 95% confidence intervals (CI) of composite severe^a^ and moderate^b^ adverse neonatal outcome^a^ among late onset FGR fetuses as per the ISUOG definition who underwent induction of labor as compared to planned cesarean delivery (reference group—planned cesarean delivery)

	*p*-value	aOR (95% CI)
Composite severe adverse neonatal outcome^a^	0.01	0.33 (0.17–0.62)
Composite moderate adverse neonatal outcome^b^	< 0.01	0.44 (0.25–0.79)

Adjusted for maternal age, nulliparity, previous cesarean delivery, obesity, fertility treatments, abnormal CPR (Cerebral Placental Ratio)

*FGR* Fetal Growth Restriction

^a^Defined as the presence of at least one of the following: perinatal death (neonatal death within 28 days), umbilical arterial pH < 7.05 or base deficit ≥ 12 mmol/L, 5-min Apgar score < 4, moderate-to-severe hypoxic-ischemic encephalopathy or receipt of therapeutic hypothermia, grade III–IV intraventricular hemorrhage, Bell stage ≥ II necrotizing enterocolitis, culture-proven neonatal sepsis or need for invasive mechanical ventilation > 24 h

^b^Defined as at least one of the following: neonatal intensive care unit (NICU) admission > 72 h, 5-min Apgar score 4–6, umbilical arterial pH 7.05–7.10, need for non-invasive respiratory support (continuous positive airway pressure or high-flow nasal cannula > 6–12 h) without intubation, transient tachypnea of the newborn, or neonatal resuscitation limited to bag-mask ventilation or brief positive-pressure ventilation in the delivery room

In adjusted analyses, IOL was associated with lower odds of invasive ventilation (aOR 0.28; 95% CI 0.15–0.54; *p* < 0.01) and prolonged NICU admission (aOR 0.48; 95% CI 0.26–0.88; *p* = 0.02), while differences in rare outcomes were not modeled due to low event counts.

Propensity score–adjusted analyses within the ISUOG-defined FGR subgroup yielded similar results. IOL remained associated with a significantly lower risk of the severe composite adverse neonatal outcome (aOR 0.32; 95% CI 0.17–0.63; *p* = 0.01) and the moderate composite outcome (aOR 0.51; 95% CI 0.29–0.90; *p* = 0.03). These findings were concordant with the primary adjusted analyses, indicating consistency across FGR definitions and analytic approaches (Supplementary Table 2).

## Discussion

### Principal findings

In this retrospective cohort study of pregnancies complicated by LOFGR, we compared neonatal outcomes between IOL and planned CD. Using both the SMFM and ISUOG definitions, IOL was associated with a significantly lower risk of both severe and moderate adverse neonatal outcomes compared with planned CD. Although approximately one in five inductions required intrapartum CD, IOL was also associated with lower rates of several individual morbidities, including prolonged NICU admission and need for mechanical ventilation.

### Results in the context of the literature

Our findings add to the ongoing debate regarding the optimal mode of delivery for LOFGR. Prior studies have yielded conflicting results. Some suggested that CD may reduce neonatal morbidity, particularly in severe cases of LOFGR, while others found no significant differences between IOL and CD. 

Baalbaki et al. evaluated 101 preterm FGR fetuses (< 34 weeks’ gestation) and found that planned CD did not confer a reduced risk of adverse neonatal outcomes, even in cases with abnormal umbilical artery Dopplers [27]. Conversely, Rodriguez-Sibaja et al. studied 212 pregnancies beyond 34 weeks and reported higher neonatal morbidity among both spontaneous labor and IOL groups compared with planned CD, largely due to a high rate of emergency CD for intrapartum distress [28]. Futterman et al. likewise observed no differences in primary outcomes between IOL and planned CD in 102 FGR pregnancies with abnormal Dopplers, although 41% of those induced ultimately required intrapartum CD [29].

By contrast, our study, which included a substantially larger population and applied contemporary SMFM and ISUOG definitions, suggests that IOL can be undertaken safely in pregnancies at > 34 weeks’ gestation and in a carefully monitored setting. Notably, despite a higher frequency of biochemical markers of acidemia, including umbilical arterial pH < 7.05 and base deficit ≥ 12 mmol/L, IOL was associated with a significantly lower incidence of the composite severe adverse neonatal outcome compared with planned cesarean delivery. The finding that IOL was associated with lower odds of composite severe neonatal morbidity is particularly noteworthy, as prior reports rarely distinguished between degrees of outcome severity. Importantly, prelabor cesarean delivery is a well-established risk factor for neonatal respiratory morbidity. The absence of labor-associated hormonal and mechanical adaptations has been linked to increased rates of transient tachypnea of the newborn, respiratory distress syndrome, persistent pulmonary hypertension, and a greater need for neonatal resuscitation and prolonged ventilatory support. These risks are inherent to the mode of delivery itself and are particularly pronounced at earlier gestational ages, likely contributing to the higher burden of respiratory morbidity observed in the planned cesarean delivery group in our cohort. The reduced risk of respiratory complications and need for resuscitation after IOL may also reflect advances in intrapartum monitoring, timely operative intervention, and neonatal care, which mitigate the risks historically attributed to trial of labor in FGR. At the same time, the persistent 20% intrapartum CD rate underscores the challenge of identifying which fetuses can tolerate labor and highlights the importance of individualized counseling.

### Clinical implications

Within the framework of modern obstetric care, IOL appears to be a reasonable and safe alternative to planned CD for LOFGR, provided that close surveillance and timely operative delivery are available. For patients, IOL offers the possibility of vaginal delivery and reduced short-term morbidity in certain domains, while avoiding the long-term implications of CD. However, because IOL carries a significant likelihood of intrapartum CD, the decision should be individualized. Maternal characteristics, fetal condition, prior obstetric history, and institutional resources must be carefully considered, and shared decision-making remains essential.

### Research implications

Further studies are needed to refine risk stratification for IOL versus CD in LOFGR. Prospective multicenter studies with standardized definitions, stratification by Doppler findings and severity of restriction, and longer-term follow-up are required. Importantly, future work should distinguish between severe and moderate neonatal outcomes, as combining them may obscure clinically meaningful differences. Research into predictors of intrapartum CD after IOL could enhance counseling and improve patient selection.

### Strengths and limitations

The strengths of this study include its large sample size, application of both SMFM and ISUOG definitions, and comprehensive, prospectively maintained electronic records, with contemporaneous ultrasound and Doppler data within 14 days of delivery. These features enhance the validity and generalizability of the findings.

Several limitations merit consideration. The retrospective design introduces potential for selection bias and residual confounding. Women undergoing planned CD were older, more often obese, and more likely to have abnormal Doppler findings, and although regression models adjusted for these factors, unmeasured confounders such as comorbidities, socioeconomic status, and provider preference may have influenced outcomes. The single-center setting may also limit generalizability. By excluding pregnancies < 34 weeks, our findings apply only to LOFGR and cannot be extrapolated to earlier, more severe disease. Finally, although we analysed severe and moderate outcomes separately, some secondary outcomes remain susceptible to practice variability, and further prospective validation is warranted.

## Conclusions

In this large retrospective cohort of pregnancies complicated by FGR at > 34 weeks’ gestation, IOL was associated with a lower risk of both severe and moderate adverse neonatal outcomes compared with planned CD. Induction was also linked to reductions in specific morbidities, including the need for mechanical ventilation, and prolonged NICU admission. However, approximately one in five women required intrapartum CD, underscoring the need for careful counseling and preparedness for operative intervention. These findings support induction as a safe and reasonable alternative to planned cesarean when conducted in settings with close intrapartum surveillance and timely access to CD. Prospective multicenter studies are warranted to validate these results, refine patient selection, and identify subgroups most likely to benefit from induction versus planned cesarean. Mode of delivery should continue to be individualized, integrating maternal characteristics, fetal condition, and institutional resources within a framework of shared decision-making.

## Supplementary Information

Below is the link to the electronic supplementary material.

Supplementary file1 (DOCX 16 KB)

## Data Availability

No datasets were generated or analysed during the current study.
