# Supplementary material for: Planned mode of delivery and neonatal outcomes in pregnancies complicated by late-onset fetal growth restriction: a retrospective cohort study
Source: Arch Gynecol Obstet. 2026 Mar 2;313(1):113. doi: 10.1007/s00404-026-08357-8 (PMC12953308; doi:10.1007/s00404-026-08357-8)
Supplement: Supplementary file 1 — Supplementary file1 (DOCX 16 KB) [file 404_2026_8357_MOESM1_ESM.docx]

**Supplementary Table 1: Propensity score-adjusted association between induction of labor and severe* or moderate** composite adverse outcome among FGR fetuses as per the SMFM definition (Adjusted OR and 95% confidence intervals (CI))**

|  | | |  |
| --- | --- | --- | --- |
|  | p-value | aOR (95% CI) | |
| Composite severe adverse neonatal outcome* | 0.01 | 0.34 (0.18-0.66) | |
| Composite moderate adverse neonatal outcome** | 0.02 | 0.51 (0.29-0.90) | |
| Adjusted for maternal age, nulliparity, previous cesarean delivery, obesity, fertility treatments, abnormal CPR (Cerebral Placental Ratio); FGR – Fetal Growth Restriction  * defined as the presence of at least one of the following: perinatal death (neonatal death within 28 days), umbilical arterial pH <7.05 or base deficit ≥12 mmol/L, 5-minute Apgar score <4, moderate-to-severe hypoxic-ischemic encephalopathy or receipt of therapeutic hypothermia, grade III–IV intraventricular hemorrhage, Bell stage ≥II necrotizing enterocolitis, culture-proven neonatal sepsis or need for invasive mechanical ventilation >24 hours.  ** defined as at least one of the following: neonatal intensive care unit (NICU) admission >72 hours, 5-minute Apgar score 4–6, umbilical arterial pH 7.05–7.10, need for non-invasive respiratory support (continuous positive airway pressure or high-flow nasal cannula >6–12 hours) without intubation, transient tachypnea of the newborn, or neonatal resuscitation limited to bag-mask ventilation or brief positive-pressure ventilation in the delivery room. | | | |

**Supplementary** **Table 2: Propensity score-adjusted association between induction of labor and severe* or moderate** composite adverse outcome among FGR fetuses as per the ISUOG definition (Adjusted OR and 95% confidence intervals (CI))**

|  | | |  |
| --- | --- | --- | --- |
|  | p-value | aOR (95% CI) | |
| Composite severe adverse neonatal outcome* | 0.01 | 0.32 (0.17-0.63) | |
| Composite moderate adverse neonatal outcome** | 0.03 | 0.51 (0.29-0.90) | |
| Adjusted for maternal age, nulliparity, previous cesarean delivery, obesity, fertility treatments, abnormal CPR (Cerebral Placental Ratio); FGR – Fetal Growth Restriction | | | |

* defined as the presence of at least one of the following: perinatal death (neonatal death within 28 days), umbilical arterial pH <7.05 or base deficit ≥12 mmol/L, 5-minute Apgar score <4, moderate-to-severe hypoxic-ischemic encephalopathy or receipt of therapeutic hypothermia, grade III–IV intraventricular hemorrhage, Bell stage ≥II necrotizing enterocolitis, culture-proven neonatal sepsis or need for invasive mechanical ventilation >24 hours.

** defined as at least one of the following: neonatal intensive care unit (NICU) admission >72 hours, 5-minute Apgar score 4–6, umbilical arterial pH 7.05–7.10, need for non-invasive respiratory support (continuous positive airway pressure or high-flow nasal cannula >6–12 hours) without intubation, transient tachypnea of the newborn, or neonatal resuscitation limited to bag-mask ventilation or brief positive-pressure ventilation in the delivery room.
